# Childhood Trauma and Self-harm in Youths with Bipolar Disorders

**DOI:** 10.2174/1570159X21666230213155249

**Published:** 2023-02-14

**Authors:** Delfina Janiri, Michelangelo Di Luzio, Silvia Montanari, Daniele Hirsch, Alessio Simonetti, Lorenzo Moccia, Eliana Conte, Ilaria Contaldo, Chiara Veredice, Eugenio Mercuri, Gabriele Sani

**Affiliations:** 1 Department of Psychiatry, Fondazione Policlinico Universitario Agostino Gemelli IRCCS, Rome, Italy;; 2 Department of Psychiatry and Neurology, Sapienza University of Rome, Rome, Italy;; 3 Department of Neuroscience, Child and Adolescence Neuropsychiatry Unit, IRCCS “Bambino Gesù” Children’s Hospital, Rome, Italy;; 4 Department of Neuroscience, Section of Psychiatry, Università Cattolica del Sacro Cuore, Rome, Italy;; 5 Early Intervention Unit, ASL Roma 3, Rome, 00152, Italy;; 6 Pediatric Neurology Unit, Fondazione Policlinico Universitario Agostino Gemelli IRCCS, Rome, Italy;; 7 Pediatric Neurology, Department of Woman and Child Health and Public Health, Università Cattolica del Sacro Cuore, Child Health Area, Rome, Italy

**Keywords:** Bipolar disorders, youth, childhood trauma, self-harm, pediatric, childhood trauma questionnaire

## Abstract

**Background:**

Bipolar disorders (BD) in youth are associated with a high risk of self-harm behaviors. Childhood trauma (CT) is a relevant environmental stressor that is related to both BD diagnosis and self-harm in adulthood. It is not yet established whether CT may impact self-harm risk in youth. Therefore, the aim of this study was to investigate the distribution patterns of CT in youth BD with and without self-harm.

**Methods:**

We assessed 273 participants (aged 13-25 years), 96 youths with BD according to DSM-5 criteria and 177 healthy controls (HC). History of CT was obtained using the Childhood Trauma Questionnaire (CTQ). The association between CT and self-harm was tested using multivariate statistical models.

**Results:**

Over 45% of participants with BD reported lifetime self-harm. The BD Self-harm group reported more emotional abuse, emotional neglect, sexual abuse, and physical abuse than HC. The BD No-Self-harm group reported more emotional abuse than HC. The BD Self-harm group reported more emotional abuse and neglect than the BD No-Self-harm group. The BD Self-harm group also reported separated parents, hospitalizations, smoking, use of antiepileptics, antipsychotics and lithium. Emotional abuse was an independent predictor of self-harm in youths with BD.

**Conclusion:**

Findings support the importance of assessing CT, in particular emotional abuse, in youth with BD at risk for self-harm.

## 1 INTRODUCTION

Self-harm rates in youths have been steadily increasing over the last decade [1, 2]. Self-harm is defined as self-poisoning or self-injury irrespective of the suicidal intent, with self-injury being the most prevalent [3]. Self-injurious behaviors include self-cutting, -burning, -biting, -hitting, and skin damage by other methods. The usual onset of self-harm is in adolescents aged around 14 years, with an estimated prevalence that is higher in girls than in males and that range between 10% and 25% in community samples [4]. Youth psychopathology is the most robust risk factor for self-harm.Previous studies found that anxiety and mood symptoms, in particular, have the highest odds ratios for both occasional and repetitive self-injury. Environmental risk factors, including family-related variables, have been also found as predictors of self-injurious behaviors.

Bipolar disorders (BD) typically present with an age at the onset that averages 15-25 years and with a strong association with an increased risk of suicide and non-suicidal self-harm [5]. Even though many studies have previously highlighted the increased suicide risk in adolescents and young adults with BD [6-8], less attention has been given so far to the relationship between self-harm and BD in youth. Recent studies indicated a lifetime self-harm prevalence in adolescents with BD ranging between 31-37% [9, 10], with a peak that occurs in the first 3 months following initial diagnosis [11].

Among early stressors, childhood trauma (CT) has emerged as one of the most important risk factors for psychiatric disorders [12], including mood disorders [13]. Distinct types of traumatic experiences (*i.e*., sexual, physical, emotional abuse and neglect) may impact psychiatric illnesses differently, modulating their clinical expression in adult age [14]. Early adverse events have been associated with biological alterations underlying mood disorders [15], as well as with negative clinical outcomes, including increased suicide risk [8, 16]. Despite the emphasis given to childhood traumatic experiences in the course of BD during adulthood, relatively little is known about youth age [17], with very few studies that are specifically focused on the clinical correlates of CT on juvenile BD [18-20]. Romero *et al.* reported that 20% percent of their sample experienced physical and/or sexual abuse [20]. Youths with CT presented with a more severe clinical course as compared with the non-abused group [20]. Marchand *et al.* [18] also highlighted that exposure to adverse events is common among pediatric patients with BD and may have a negative impact on prognosis. They specified that 53% of youths with BD experienced childhood maltreatment. A similar prevalence was observed by Cazala and colleagues [19], who found that the severity of CT types was from low to moderate and that more than 50% of individuals with pediatric BD reported having experienced emotional abuse.

Interestingly, two studies found a significant impact of early adverse events on suicide risk [19, 21]. Specifically, Cazala *et al.* [19] showed that females with BD and histories of physical abuse were more likely to report suicidal thoughts and behaviors than the rest of the sample. Goldstein *et al.* [21] reporting data on over 400 children and adolescents aged 7-17 year, found that youths with a lifetime history of suicide attempt were more likely to present with physical and/or sexual abuse than non-attempters.

However, no study to date has focused on the relationship between self-harm and CT in youths with BD. Accordingly, the prime aim of our study was to fill this gap by investigating the link between self-harm and CT in a large sample of adolescents and youths with BD compared to a group of healthy controls (HC). We were specifically interested in identifying distribution patterns of different CT subtypes in patients with and without self-harm. We hypothesized that self-harm in youth is associated with early adverse events. Additionally, we also investigated whether childhood traumatic experiences and familial characteristics may differently impact self-harm behaviors in youths with BD.

## 2 MATERIALS AND METHODS

### 2.1 Participants

For the primary aim of the study, we focused on the prevalence of self-harm in patients with bipolar disorders (BD) and on its relation with childhood trauma (CT). Given that BD typically presents with an age at onset that averages 15-25 years, we considered patients aged 13-25, in order to include teenage age and to encompass BD onset. Additionally, to avoid biases in identifying distribution patterns of different CT subtypes in patients with and without self-harm, we applied the inclusion/exclusion criteria described below. We consecutively assessed 96 young outpatients with a DSM-5 diagnosis of BD type I, II or unspecified. Patients were enrolled at the Early Intervention for Mood Disorders Unit at Fondazione Policlinico Universitario Agostino Gemelli IRCCS in Rome, Italy. Patients were screened by trained staff for DSM-5 disorders, and clinical diagnoses were confirmed using the Structured Clinical Interview for DSM-5-Research Version [15]. In addition to a diagnosis of BD, inclusion criteria were as follows: (i) age between 13 and 25 years, in order to include teenage age and to encompass BD onset [22], (ii) stable phase of illness according to psychometric evaluation (Hamilton Depression Rating Scale, HAM-D ≤ 7; Young Mania Rating Scale ≤ 12), (iii) fluency in Italian, and (iv) at least five years of school education. Exclusion criteria were as follows: (i) traumatic head injury with loss of consciousness; (ii) lifetime history of major medical or neurological disorders; (iii) cognitive impairment; (iv) recent (past six weeks) changes in any psychotropic medication; (v) current use of stimulant medications; and (vi) a history of psychosis unrelated to the primary mood disorder. We also recruited 117 healthy controls (HC), matched for age, sex, and educational level, from the same geographical area. All HC were screened for current or lifetime history of DSM-5 disorders. For the aims of this study, they were also interviewed to determine their potential self-harm behaviors; none of them reported lifetime self-harm behaviors. Participants were also interviewed to assess whether any first-degree relative was affected by mood disorders or schizophrenia. If they had a positive family history, they were excluded. Other exclusion criteria were the same as those for the patient group. The study was approved and undertaken in accordance with the guidelines of the Fondazione Policlinico Universitario Agostino Gemelli Ethics Committee and in accordance with the Principles of Human Rights, as adopted by the World Medical Association at the 18^th^ WMA General Assembly, Helsinki, Finland, June 1964 and subsequently amended at the 64^th^ WMA General Assembly, Fortaleza, Brazil, October 2013. All participants and their parents, if patients < 18 years old, gave their written informed consent to participate in the study after they had received a complete explanation of the study procedures.

### 2.2 Clinical Assessment

We used the short form of the Childhood Trauma Questionnaire (CTQ) to measure early adverse childhood events. This is a 28-item, retrospective, self-report questionnaire [23] that investigates traumatic experiences in childhood. The questionnaire assesses five types of CT: emotional abuse, emotional neglect, physical abuse, physical neglect and sexual abuse. For each type of trauma, scores range from 5 to 25. Higher scores indicate greater childhood maltreatment. The CTQ has been used in youths in both non-clinical [24] and clinical populations [19] with a high degree of reliability.

Clinical characteristics were collected during a clinical interview. Lifetime self-harm was assessed with a semi-structured questionnaire consisting of two parts, one related to the past 6 months and the other lifetime. Each part included two questions: [1] “Have you ever intentionally (*i.e*., on purpose) performed each type of self-harm (*i.e*., self-cutting, biting, banging, hitting, burning, self-poisoning) [2] Have you ever performed each type of self-harm without suicidal intent (*i.e*., not for suicidal reasons)? Respondents had to answer only “Yes” or “No”. If they answered “yes” to both the questions listed above, they were considered in the self-harm group. The wording of the questions can be changed to improve/ check understanding and the final evaluation was also based on information from parents (always present during visits) and from any medical documentation. All demographic, familial and clinical data collected were entered in preprinted medical records.

### 2.3 Statistical Analyses

We compared the three groups' (*i.e*., BD youths with and without self-harm, and HC) socio-demographic and clinical characteristics on the basis of the chi-square test for nominal variables and one-way analysis of variance (ANOVA) followed by post-hoc Scheffé tests for continuous variables and by pairwise post-hoc analyses for nominal variables. Significance was set at a *p* < 0.05 level.

For the first aim of this study, we focused on the distribution patterns of CT subtypes. First, we conducted a series of multivariate analyses of variance (MANOVA) using all the CT subtypes as dependent variables and the diagnostic groups (*i.e*., BD No Self-harm, BD Self-harm and HC) as an independent factor. When the initial model was significant, we conducted a series of one-way ANOVAs, followed by Scheffé post-hoc tests, to compare means among groups. For the ANOVA comparative measurements, we used a statistical model corrected for multiple comparisons according to the Bonferroni procedure (*p* < .05/number of comparisons) to minimize the likelihood of type I statistical errors. For the secondary aim of the study, we focused on which variables significantly contributed to differentiate youth with and without self-harm among childhood traumatic experiences and familial characteristics. Accordingly, we performed a multivariate logistic regression model considering self-harm as the dependent variable and CT subtypes, number of cohabitants and having divorced parents as independent variables together with age, sex and significant clinical variables associated with self-harm in the univariate analyses. Significance was set at a *p* < 0.05 level. All statistical analyses were performed using SPSS v. 25 (IBM Corp., USA).

## 3 RESULTS

### 3.1 Sociodemographic and Clinical Characteristics

The final sample included 273 individuals, 211 females (77.3%) and 62 males (22.7%), with a mean age of 18.51 (SD = 3.41) years. In the overall group of patients with a diagnosis of BD (n = 96), 44 (45.8%) youths reported non-suicidal lifetime self-harm (BD-SH) and 52 did not report self-harm (BD-NSH). The three diagnostic groups (*i.e*., BD-NSH, BD-SH and HC) did not differ significantly for age, gender or educational level. The three group significantly differed for number of cohabitants, having divorced parents and smoking status. Specifically, post-hoc analyses clarified that HC reported higher number of cohabitants then BD-NSH (*p* = 0.01); both BD-SH (Chi-square = 5.88, df = 1, *p* = 0.003) and BD-SH (Chi-square = 14.51, df = 1, *p* < 0.001) presented with higher percentage of separated parents than HC; BD-NSH reported less smoking use than BD-SH (Chi-square = 9.97, df = 1, *p* = 0.002) and HC (Chi-square = 9.17, df = 1, *p* = 0.002) groups (Table **1**).

With respect to clinical characteristics, BD-SH reported more hospitalization, and more use of antiepileptics, antipsychotics and lithium than BD-NSH (Table **1**).

### 3.2 Distribution Patterns of Childhood Trauma Subtypes in Patients with and Without Self-harm

A preliminary MANOVA found a significant global effect (Wilk's Lambda = 0.72, F = 9.11, df = 10, *p* < 0.0001) of CTs on the presence of lifetime self-harm (categorized as YES/NO). Factorial ANOVAs indicated a main effect for all types of CT, except psychical neglect, which did not survive multiple comparison correction. In particular, a series of pairwise Scheffé post-hoc analyses clarified that BD-SH reported more emotional abuse, emotional neglect, sexual abuse, and physical abuse than HC, as well as more emotional abuse and neglect compared to BD-NSH. In addition, BD-NSH reported more emotional abuse than HC (Table **2**).

### 3.3 Effect of Childhood Traumatic Experiences and Familial Characteristics on Self-harm

In the multivariate logistic regression only emotional abuse, among childhood, traumatic experiences and familial characteristics significantly predicted self-harm during lifetime (*p* = 0.008). Specifically, increasing emotional abuse during childhood was associated with an increased likelihood to present with self-harm (OR = 1.30, 95% CI = 1.07-1.58, Wald = 7.01).

## 4 DISCUSSION

To our knowledge, this is the first study investigating the relationship between self-harm and childhood trauma (CT) in youths with bipolar disorders (BD). Results showed that around 45% of youths with BD reported having performed nonsuicidal self-harm during lifetime. Youths with BD and self-harm reported more emotional abuse, emotional neglect, physical abuse and sexual abuse than HC, while only emotional abuse differentiated patients without self-harm from HC. When we directly compared BD with and without self-harm, we observed significant differences only in emotional abuse and emotional neglect. Notably, after modelling CT and familial characteristics, emotional abuse showed the most consistent association with self-harm in BD.

The distribution of childhood trauma in youths appears in line with previous findings in adults, where CT was associated with both BD diagnosis and life time self-harm/suicidal behaviors [25]. In adulthood, self-harm seems to be correlated with a high frequency of early adverse events, in particular sexual abuse, *per se* [26]. In parallel, the link between BD diagnosis and CT has been solidly established, with emotional abuse being the most frequent type of early adverse event in adult patients with BD [13]. In the youth population, BD and self-harm presented together with a prevalence ranging between 31-37% [9, 10], slightly smaller than the one we found in the current study. In parallel, a significant association was recently found between CT and BD diagnosis in youth [19]. Furthermore, a large population-based cohort study recently showed that adverse childhood experiences were associated with increased self-harm risk in adolescents [27]. Here, for the first time, we specified that the co-occurrence of the two conditions (*i.e*., BD diagnosis and CT) might be particularly at risk for the development of self-harm behaviors in youth. Future longitudinal studies are needed to confirm these initial observations.

In our study, we found higher emotional abuse in BD without self-harm compared to HC, suggesting emotional abuse as a characteristic trait of BD in youth. The evidence that emotional abuse is particularly linked to BD is consistent with previous findings in adults highlighting that this is the most frequent type of childhood traumatic experiences in patients with BD [13]. Meta-analytic findings confirmed these results, showing that emotional abuse was four times more likely to occur in BD than in HC, a larger effect than for other types of early adversities [13]. Furthermore, a recent study found that childhood maltreatment, and emotional abuse, in particular, may increase the risk to develop BD even when the genetic load is low [28]. Our study seems to suggest that these observations may be extended to adolescents and youths with BD. Future studies with larger samples may confirm this initial hypothesis.

It is noteworthy that in our study, when the two BD groups were compared (*i.e*., BD with and without self-harm), we found significant differences only in emotional abuse and emotional neglect. Furthermore, after modelling CT and familial characteristics, emotional abuse showed the strongest association with self-harm. These results are consistent with previous studies in adults showing a specific association between emotional abuse and suicidal behaviors in patients with mood disorders [16]. They are also in line with recent neuroimaging studies separately investigating neural circuits implicated in BD, childhood abuse and self-harm. A very recent study found altered resting-state functional connectivity between amygdala and the dorsolateral and orbitofrontal cortex in adolescent BD with self-harm [29]. Interestingly, the same brain areas have also been implicated in the neurobiological effects of childhood abuse in adulthood [30]. In particular, in a previous study, we found that CT in BD has a specific effect on volumetric abnormalities in the amygdala [15]. Intriguingly, a recent systematic review found that amygdala hyperactivity to emotional stimuli was the most commonly reported finding in youth with BD compared to HC [31]. Furthermore, hyperactivity to emotional stimuli has been separately reported in both patients with BD and emotional abuse [32] and in patients with self-harm [33]. Even though no neuroimaging study is available to date on CT in youth BD, we could speculate that specific neural underpinnings may be shared between self-harm and CT, in particular emotional abuse. These early speculations may be used to plan future neuroimaging studies in youths with BD, as well as to tailor prevention and intervention strategies for self-harm risk.

Considering the effect of familial characteristics on self-harm, we found a higher percentage of separated parents in the BD Self-harm than in BD No-Self-harm group. Results are in line with previous community-representative studies showing parental separation or divorce as a risk factor for suicidal behaviors and self-harm in adolescents [34, 35]. Nevertheless, after modelling CT and familial characteristics in the logistic regression analysis, only emotional abuse resulted in a significant predictor of self-harm in youths with BD. Interestingly, previous authors attempted to frame emotional child abuse in the context of parental separation or divorce [36]. It is possible that when parents are in open conflict children may develop harmful reactions and behaviors in response to a high degree of psychological burden [36]. Accordingly, high levels of family conflict are linked to suicidal behaviors in pediatric age [8]. Further investigations are needed to clarify the relationship between parental separation/family conflict, emotional abuse and self-harm in youths with BD.

Our results showed that self-harm is associated with more hospitalization, more smoking, more use of antiepileptics, antipsychotics and lithium. These findings are in line with previous studies highlighting increasing rates of hospital-treated self-harm among young people and with the need of more complex pharmacological treatments patients with BD presenting with a severe clinical course [37, 38].

In this regard, our study corroborates the literature asking for targeted interventions in key transition stages for young people at risk for self-harm.

Before presenting our conclusions, we must acknowledge some points that might limit the generalizability of our results. First, the cross-sectional design of our study limits our ability to investigate a causal relationship between the assessed variables. Second, history of lifetime self-harm was assessed using a not validated, semi-structured questionnaire. Therefore, it is possible that the instrument we used might not be sufficiently sensitive in detecting self-harm risk. In particular, it did not provide a quantitative measure of the risk. Furthermore, it did not provide information on the type and the frequency of self-harm. This is a potential shortcoming because different type of self-harm patterns may be differently associated with distinct type of CT. Third, the reliability of the retrospective assessment of childhood traumatic experiences, as assessed with the CTQ, may be influenced by uncontrolled recall bias. Nevertheless, the CTQ is currently used in youths [19, 24] in both non-clinical [24] and clinical populations [19] and it is indicated as the best instrument for evaluating CT in BD [39]. Fourth, in our study females were overrepresented than males, accounting for over 70% of the total sample. Because the prevalence of self-harm in community samples is higher in females than in males, this imbalance may have influenced the high percentage of youths presenting with self-harm. Nevertheless, the three diagnostic group (*i.e*., BD-Self-ham, BD-No-Self-harm and HC) were balanced for gender (*p* > 0.05). Fifth, in our study we only compared individuals with BD to HC. Previous studies demonstrated a strong association between CT and other psychiatric diagnoses [40-43]. Accordingly, patients with BD and CT might be compared not only to HC but also to other diagnostic categories. Future studies may further focus on this specific point. Finally, despite the relatively large number of recruited patients, due to splitting our samples according to diagnostic group, the final samples were relatively small.

## CONCLUSION

In conclusion, our study can be considered as a first step towards a better understanding of the relationship between BD diagnosis, CT and self-harm in youth. The results of our study indicate the importance of assessing CT in youth with BD at risk for self-harm. Indeed, this is important for both research and clinical purposes. First, CT might unveil slight differences between youths with BD, that are normally difficult to identify. Individuals with CT might represent a subgroup of patients, who may present with more severe clinical outcomes and with different life-course biological trajectories. Future investigations with prospective longitudinal designs could confirm this initial speculation. Second, the relationship between self-harm and CT in youths has obvious clinical relevance. All youths with BD who have undergone CT, in particular emotional abuse, should be treated with particular attention because of their possible self-injury behaviors. The routine assessment of CT might allow an early identification of patients at risk for self-injurious behaviors even before the clinical onset of symptoms. This would allow to implement specific prevention strategies. Psychotherapeutic and psychoeducational programs purposely designed to the elaboration of traumatic events may be useful in enhancing resilience process for self-harm risk. Furthermore, the use of pharmacological treatments specifically targeting hyperreactivity to emotional stimuli might be useful in treating self-injurious behaviors in patients with BD and a history of CT.

The assessment of early trauma should be definitely included in the clinical evaluation of youths with BD to better tailor prevention and intervention strategies for self-harm risk.

## Figures and Tables

**Table 1:** Sociodemographic and clinical characteristics of BD-SH, BD-NSH and HC (N = 273).

**Characteristics**	**BD-SH (n = 44)**	**BD-NSH (n = 52)**	**HC (n = 177)**	***F* or *χ*^2^**	**df**	***P***
Age (years): mean ± (SD)	17.45 (3.31)	18.63 (3.72)	18.74 (3.32)	2.567	2	0.079
Gender, males: n (%)	6 (13.6)	13 (25.0)	43 (24.3)	2.472	2	0.291
Educational level (years): mean ± (SD)	12.07 (2.98)	12.92 (2.92)	13.17 (3.05)	2.356	2	0.097
Number of cohabitants: mean ± (SD)	3.74 (0.89)	3.50 (0.97)	3.98 (0.89)	5.081	2	0.007
Separated parents: n (%)	23 (52.3)	23 (44.2)	41 (23.2)	18.274	2	< 0.001
Smoking: n (%)	16 (36.4)	5 (9.6)	54 (30.5)	17.895	2	< 0.001
Family history of psychiatric disorders: n (%)	24 (54.5)	23 (44.2)	-	1.015	1	0.314
Age at onset (years): mean ± (SD)	14.11 (2.40)	15.41 (4.36)	-	2.473	1	0.120
Hospitalizations: n (%)	15 (34.1)	6 (11.5)	-	7.093	1	0.008
Use of THC	11 (25.0)	6 (11.5)	-	2.964	1	0.085
**Treatments**						
Antidepressants: n (%):	8 (18.2)	15 (28.8)	-	1.488	1	0.223
Antiepileptics: n (%)	30 (68.2)	23 (44.2)	-	5.529	1	0.019
Antipsychotics n (%)	21 (47.7)	7 (13.5)	-	13.545	1	< 0.001
Lithium: n (%)	20 (45.5)	4 (7.7)	-	18.126	1	< 0.001
Benzodiazepines: n (%)	10 (22.7)	8 (15.4)	-	0.843	1	0.358
Psychotherapy: n (%)	22 (50.0)	19 (36.5)	-	1.765	1	0.184

**Abbreviations**: BD-SH = Bipolar Disorder-Self-harm; BD-NSH = Bipolar Disorder-Non-Self-Harm; HC = Healthy Controls; SD = Standard deviation; df = Degrees of freedom.

**Table 2:** Distribution patterns of childhood trauma subtypes in BD-SH, BD-NSH and HC (N = 273).

**-**	**SH mean ± (SD)**	**NSH mean ± SD)**	**HC mean ±** ** SD)**	***F* or *χ*^2^**	**df**	***P***	**HC *vs*. ** **BD-SH (*P*)**	**HC *vs*. ** **BD-NSH (*P*)**	**BD-SH *vs*. ** **BD-NSH (*P)***
Emotional abuse	14.50 (5.80)	10.14 (4.31)	8.08 (3.54)	43.37	2	< 0.001	< 0.001	0.008	< 0.001
Physical abuse	7.32 (3.41)	6.29 (2.23)	5.68 (2.32)	7.81	2	< 0.001	0.001	0.305	0.142
Sexual abuse	6.82 (3.17)	6.27 (2.58)	5.41 (2.26)	6.76	2	0.001	0.004	0.095	0.570
Emotional neglect	13.68 (3.43)	12.10 (2.88)	11.96 (2.47)	7.16	2	0.001	0.001	0.951	0.019
Physical neglect	10.00 (1.98)	9.82 (1.89)	9.37 (1.53)	3.24	2	0.041	0.088	0.242	0.878

**Abbreviations**: BD-SH = Bipolar Disorder-Self-harm; BD-NSH = Bipolar Disorder-Non-Self-Harm; HC = Healthy Controls; SD = Standard deviation; df = Degrees of freedom. **Note**: *p significant after Bonferroni correction.

## LIST OF ABBREVIATIONS

BD: Bipolar Disorders
CT: Childhood Trauma
CTQ: Childhood Trauma Questionnaire.
HC: Healthy Controls
MANOVA: Multivariate Analyses of Variance
